# Protective Effects of Protegrin in Dextran Sodium Sulfate-Induced Murine Colitis

**DOI:** 10.3389/fphar.2019.00156

**Published:** 2019-02-28

**Authors:** Evanna Huynh, Jenna Penney, Jeff Caswell, Julang Li

**Affiliations:** ^1^Department of Animal Biosciences, Ontario Agricultural College, University of Guelph, Guelph, ON, Canada; ^2^Department of Pathobiology, Ontario Veterinary College, University of Guelph, Guelph, ON, Canada; ^3^Department of Life Science and Engineering, Foshan University, Foshan, China

**Keywords:** protegrin 1, colitis and dextran, antimicrobial peptide, mouse model, inflammation

## Abstract

Cathelicidins, a class of antimicrobial peptides, have been widely studied for their antimicrobial role in innate immune responses during infection and inflammation. At sub-antimicrobial concentrations, various cathelicidins from different species have been reported to exert chemotactic activity on neutrophils, monocytes, dendritic cells and T-cells, and also enhance angiogenesis and wound healing. To date, the role of the pig cathelicidin, protegrin-1 (PG-1), in immune modulation and tissue repair in the intestinal tract has not been investigated. The aim of the present study was to examine the potential protective effects of recombinant PG-1 in a mouse dextran sodium sulfate (DSS)-induced colitis inflammation model. This is the first report showing the protective effects of PG-1 in its various forms (pro-, cathelin-, and mature-forms) in attenuating significant body weight loss associated with DSS-induced colitis (*p* < 0.05). PG-1 treatment improved histological scores (*P* < 0.05) and influenced the gene expression of inflammatory mediators and tissue repair factors such as trefoil factor 3 (TFF3) and mucin (MUC-2). Protegrin treatment also altered the metabolite profile, returning the metabolite levels closer to untreated control levels. These findings lay the foundation for future oral application of recombinant PG-1 to potentially treat intestinal damage and inflammation.

## Introduction

Inflammatory bowel disease (IBD) is characterized by inflammation of the intestinal tract that can ultimately lead to decreased epithelial barrier function. In clinical IBD studies, mucosal healing as assessed by endoscopy has emerged as a main treatment goal in IBD rather than just an indicator of successful resolution of intestinal inflammation (Neurath and Travis, 2012). Intestinal mucosal repair is an orchestrated process requiring the integration of events involving inflammation, formation of blood vessels, and tissue regeneration. Cathelicidin is a class of antimicrobial peptides (AMPs) endogenously present in epithelial and immune cells with potent antibacterial, antiviral, and antifungal functions (Bals, 2000). During infection and inflammation, expression of cathelicidin has been shown to increase when compared to control samples (Hase et al., 2002, 2003; Wong et al., 2011) In IBD patients, the colonic expression of cathelicidin is altered, with increased expression in ulcerative colitis patients compared to the non-inflamed control mucosa, suggesting this peptide may have a role in modulating intestinal inflammation (Schauber et al., 2006). Cathelicidins have been widely studied due to their antimicrobial role in innate immune responses. It is certain that neutrophil granules contain sufficiently high concentrations of AMPs to cause direct antimicrobial activity (Ganz, 1987). However, at sites such as mucosal surfaces, it is unclear whether direct antimicrobial activity of AMPs occurs, as they may be abrogated by serum and divalent cations when produced and secreted at lower concentrations by epithelial cells (Ganz, 2003; Bowdish et al., 2006). At sub-antimicrobial concentrations, various cathelicidins from different mammals have been reported to exert chemotactic activity on neutrophils, monocytes, dendritic cells, and T-cells (Verbanac et al., 1993; Scott et al., 2002; Tjabringa et al., 2003; Kurosaka et al., 2005). Furthermore, cathelicidins have been shown to enhance angiogenesis and wound healing (Zanetti, 2005; Wuerth and Hancock, 2011; Yeung et al., 2011).

In the context of intestinal tissue repair, cathelicidins have been shown to have therapeutic potential for IBD. In experimental acute dextran sodium sulfate (DSS)-induced colitis, mice deficient in the mouse cathelicidin-related antimicrobial peptide (mCRAMP-/-), developed more severe colitis compared to wild type mice (Koon et al., 2011). In the same study, the anti-inflammatory effect of mCRAMP was demonstrated in DSS-induced colitis (Koon et al., 2011). Using the same model, intracolonic administration of mCRAMP-encoding plasmid and peptide alleviated colitis in knockout mice (Tai et al., 2013).

To date, the role of the pig cathelicidin, protegrin-1 (PG-1), in immune modulation and tissue repair in the intestinal tract has not been investigated. Lung ectopic expression of PG-1 in mice has been demonstrated to enhance resistance to bacterial infection, potentially by modulating inflammatory responses (Cheung et al., 2008). Although protegrin is from the cathelicidin family of AMPs, it has a β-hairpin structure in contrast to the α-helical peptides of mouse and human cathelicidin (Kościuczuk et al., 2012). As a result, improved understanding of different cathelicidins, including protegrin, may further define the therapeutic potential of cathelicidin in IBDs. Furthermore, the evolutionarily conserved pro-region of cathelicidins (the precursor cathelin domain) has been suggested to have a secondary purpose such as protease-inhibition and antimicrobial activity once proteolytically cleaved from the antimicrobial mature peptide (Zaiou et al., 2003; Zhu et al., 2008; Andrault et al., 2015). In the present study, we examined the effects of proform PG-1 (ProPG), the cathelin domain (cathelin), and mature-form PG-1 (mature PG-1) produced by *P. pastoris* in a mouse model of DSS-induced colitis.

## Materials and Methods

### Animals and Induction of Colitis

The procedures for use of animals in this study were in accordance with the guidelines of the Canadian Council for Animal Care, and all work was approved (AUP3470) by the University of Guelph Animal Care Committee. The study comprised five groups of mice. Four groups received DSS in the drinking water to induce colitis and were treated once daily as follows: mature PG-1 (PG-1 + DSS), pro-form PG (ProPG + DSS), the cathelin domain (cathelin + DSS), PBS (PBS + DSS). In addition, one group did not receive DSS and was treated with PBS. Mature PG-1 refers to the portion of the peptide that is left once the cathelin domain is cleaved off, Pro-PG is the full-length protein consisting of Mature PG-1 + the cathelin domain. A total of 60 mice (12 per group) were used in the study. Male and female (50:50) Balb/c (Charles River) mice (5-weeks-old) were used in the experiments. All mice were housed in a temperature-controlled environment with a 12-h light and 12-h dark cycle and provided free access to water and 14% protein rodent maintenance diet (2014 Teklad global standard, Evigo). Mice were treated via intragastric gavage through sterile animal feeding needles once daily (10 AM) for 10 consecutive days with 10 mg/kg body weight (BW) of recombinant mature PG-1, cathelin, proform PG-1 (ProPG), or the same volume of PBS. Acute colitis was induced according to published protocols (Qiu et al., 2013) with minor modifications by 5% dextran sulfate sodium (DSS) (molecular weight, 35–50 kDa; MP Biomedical, United States) dissolved in drinking water for 10 days. Fresh DSS solution was provided to the mice daily.

### Disease Activity Index Assessment

All animals were daily examined and the disease activity index (DAI) score was assessed as previously described (Maxwell et al., 2009) by assessing stool consistency, presence of blood in the stool and BW (summarized in Table 1). The percentage of BW loss was calculated relative to the initial BW (Day 0) using the following method: [(Weight on day X-Initial weight)/Initial weight] × 100 (Maxwell et al., 2009).

**Table 1:** Disease activity index (DAI) scoring system for mice with DSS-induced colitis (Marchesi et al., 2007).

Disease activity index (DAI)			
**Score**	**Stool consistency**	**Stool blood**	**% Body weight loss**

0	Normal	Normal	0–1%
1	Moist and sticky	Brown reddish color	1–5%
2	Soft	Visible blood	5–10%
3	Diarrhea	Rectal bleeding	10–20%

### Examination of Intestinal Morphology

The mice were euthanized using carbon dioxide at day 10 of treatment. The small and large intestines were straightened for length measurements and emptied for weight measurements. All data were collected in a blind manner. Approximately 0.5 cm of each colon was isolated, rinsed with sterile PBS, and fixed overnight with 10% formalin. Fixed tissues were embedded in paraffin, sectioned (5 μm), and stained with hematoxylin and eosin (H&E) for double-blind morphologic examination. Three cross sections of each of the intestinal segments were viewed. In each cross-sectioned tissue sample, 10–15 complete villus-crypt structures were examined microscopically for goblet cell count. Mucosal damage was assessed based on previously described criteria (Araki et al., 2006): by histologically scoring surface epithelial loss, inflammatory cell infiltration into the mucosa, and crypt destruction (0 for no change; 1 for localized and mild, 2 for localized and moderate; 3 for extensive and moderate; 4 for extensive and severe). The mucosal damage score was the sum of these three parameters for a maximum possible score of (12).

### Reverse Transcription Quantitative PCR (RT-qPCR)

To assess the gene expression profile in the colon, RNA was isolated from formalin-fixed tissues according to the manufacturer’s instruction indicated in the FFPE RNA purification kit (Norgen Biotek Corporation, Canada). Cross sections of approximately 0.5 cm were used. Total RNA was isolated and then treated by RNase-free DNase I. The first-strand complementary DNA (cDNA) was synthesized according to manufacturer’s instruction. PCR was performed using Mx3005 cycler (Stratagene, CA, United States) with SYBRPremix Ex Taq (Takara Clontech, Otsu, Japan). Each PCR reaction was performed in duplicate, with water controls, in which DNA template was replaced with water to monitor potential template contamination in the assay, under the following conditions: denaturation at 95°C for 10 min, three-step amplification including denaturation at 95°C for 15 s, annealing at 60°C for 30 s, extension at 72°C for 30 s and a subsequent melting curve (55–95°C) determination with continuous fluorescence measurement and final cooling to room temperature. Primer sequences were as follows:

Mouse mucin 2 (Muc-2): Forward (5′ CCCAGAAGGGACTGTGTATG 3′) and reverse (5′-TGCAGACACACTGCTCAC A-3′); mouse trefoil factor 3 (TFF3): Forward (5′ TAATGCTGTTGGTGGTCCTG 3′) and reverse (5′-CAGCCACGGTTGTTACACTG-3′); mouse cyclooxygenase 2 (Cox-2): Forward (5′ ATGATCTACCCGCCTCACAC 3′) and reverse (5′GCAGCTCTGGGTCAAACTTC3′); mouse Tumor necrosis factor alpha (TNFα): Forward (5′ GCCTCTTCTCATTCCTGCTTG′) and reverse (5′CTGATGAGAGGGAGGCCATT′); mouse β-actin: Forward (5′ TGACTGACTACCTCATGAAGATCC-3′) and reverse (5′TCTCCTTAATGTCACGCACGATT-3′). Relative mRNA levels were determined using the comparative Ct method (Schmittgen and Livak, 2008) and β-actin gene was used as the reference gene.

### Serum Sample Preparation and Metabolomic Analysis

Blood was collected via cardiac puncture after euthanasia into 1.5 mL Eppendorf tubes and stored at room temperature for clotting (30 min). Samples were centrifuged at 1500 × *g* for 10 min at 4°C. Serum aliquots of approximately 50–100 μl were then transferred into sterile cryovials, frozen and stored at -80°C. Sample preparation and metabolomic analyses were performed at The Metabolomics Innovation Centre (TMIC), University of Alberta, Canada. Untargeted quantitative metabolomics using a combined Direct Flow Injection (DFI-) and liquid chromatography (LC-) MS/MS assay was used for the metabolomic analyses of the samples.

### Statistical Analysis

Results are expressed as mean ± SEM (standard error of the mean). The data were analyzed by two-factor analysis of variance (ANOVA) using Prism version 5.0 analysis software (GraphPad Software). Data sets were analyzed by Tukey’s test for multiple comparisons to determine statistical differences between groups. The results were considered significant at a *P-*value of <0.05.

Statistical comparisons of data between treatment and controls of the *metabolomic analysis* data were performed using univariate ANOVA, or Fisher exact test, as appropriate with MetaboAnalyst 3.0, a comprehensive tool suite for metabolomic data analysis^1^.

## Results

### Effect of Protegrin on Body Weight and Disease Activity Index (DAI) in Colitis

**FIGURE 1:**
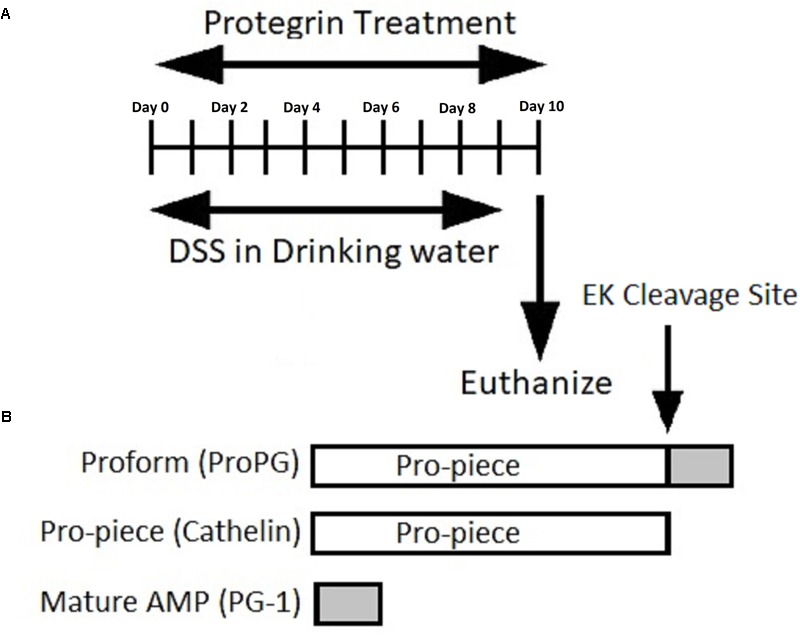
**(A)** Timeline representation of induction of colitis and protegrin treatment. **(B)** Schematic representation of the recombinant protegrin and its various forms: pro-, cathelin-, and mature-form PG-1.

**FIGURE 2:**
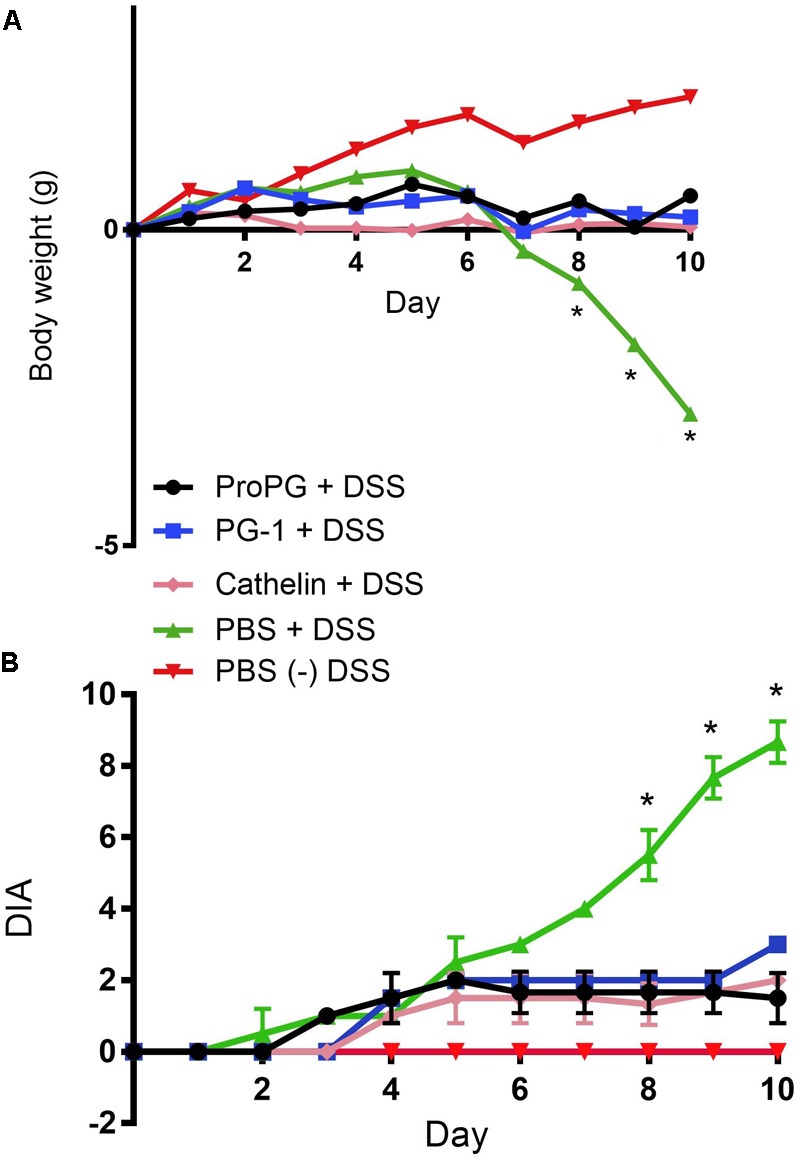
Protegrin treatments ameliorate weight loss and disease activity index (DAI) in DSS-induced colitis in mice. By day 8, mice administered DSS and treated with protegrin [pro-form (ProPG) Cathelin or mature PG-1] have significantly (^∗^*P* < 0.05) significantly less weight loss **(A)** and improved total DAI scores **(B)** compared to the mice administered DSS but given PBS instead of protegrin (PBS + DSS). PBS: mice not administered DSS, treated with PBS. Higher DAI score indicates more severe disease signs. Significance was determined using a one-ray ANOVA with a *post hoc* Tukey test. Data represent the mean ± SEM of 12 mice per treatment.

The DSS-induced colitis mouse model is commonly used to study the pathogenesis and intervention methods of IBD (Vowinkel et al., 2004; Yan et al., 2009). We induced experimental colitis in Balb/c mice by adding 5% DSS to the drinking water for 10 days with and without intragastric treatment with pro-, cathelin- or mature-forms of protegrin (proPG + DSS, cathelin + DSS, and PG-1 + DSS, respectively), compared to mice administered DSS and treated with PBS (PBS + DSS) or mice not administered DSS (PBS) (Figure 1). Significant weight loss from initial (Day 0) BW was evident in the PBS + DSS group when compared to the healthy negative control, administered PBS (Figure 2A), suggesting that DSS effectively induced colitis. In groups treated with DSS and protegrin, the BW of the mice did not significantly increase or decrease from Day 0 throughout the experiment, while the BW of the mice treated with DSS without protegrin (PBS + DSS) decreased significantly starting from day 8 to the end of the trial (Figure 2A). The results suggest protegrin treatment may prevent BW loss associated with DSS-induced colitis. To further assess the severity of colitis, combined average daily scores of stool consistency, stool bleeding and BW loss were used to generate the disease activity index (DAI; Table 1). A higher DAI score indicates more severe disease signs. The DAI score remained at 0 throughout the trial in the healthy (PBS) control group (Figure 2B). By Day 8–10, the DAI score was significantly lower in protegrin-treated groups (ProPG + DSS, Cathelin + DSS, PG-1 + DSS) than in the PBS + DSS group. By Day 10, the PBS + DSS group exhibited the highest DAI score (8.6) in contrast to the scores (∼2) in the protegrin-treated groups.

### Effect of Protegrin on Intestinal Length and Weight in Colitis

**FIGURE 3:**
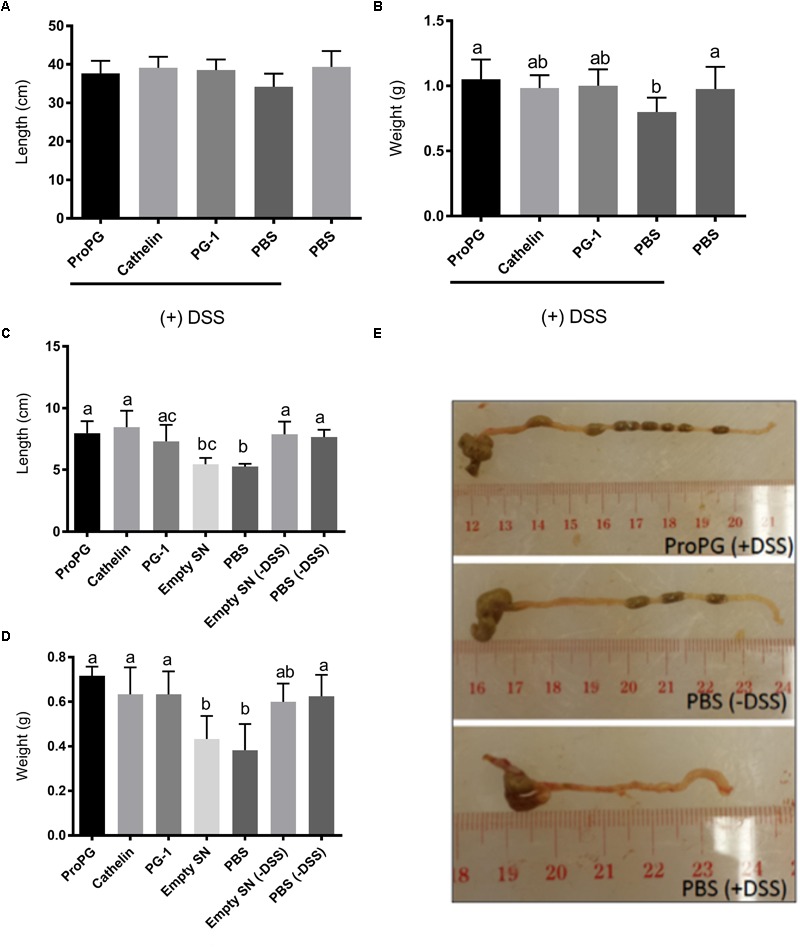
Influence of protegrin treatment groups on intestinal length and weight. **(A)** Small intestine length. **(B)** Small intestine weight. **(C)** Colon length. **(D)** Colon weight. **(E)** Representative images of colon sections. Data represent the mean ± SEM of six mice per treatment. Different letter superscripts denote significant difference between groups (*P* < 0.05 calculated using a one-way ANOVA with a *post hoc* Tukey test). Empty SN: negative control fermentation supernatant from yeast harboring the empty expression vector (the backbone vector without the coding sequences for various form of PG-1).

Dextran sodium sulfate did not have an effect on the small intestine length (Figure 3A). However, the small intestine weight was significantly reduced in the PBS + DSS group compared to the healthy PBS group (Figure 3B). Of the three forms of protegrin, only the full-length proform PG-1 (ProPG) in the ProPG + DSS group significantly prevented the small intestinal weight loss associated with DSS compared to the PBS + DSS group (*P* < 0.05; Figure 3B). The small intestinal weight of the ProPG + DSS group was not significantly different from the healthy PBS control group (*P* > 0.05), suggesting ProPG may ameliorate the negative effect of DSS on small intestinal weight.

Another measurement of the severity of colitis is the colonic length (Vowinkel et al., 2004; Yan et al., 2009). As shown in Figure 3C,D, the colon length was shortened by 30% and the colonic weight decreased by 33% in DSS-treated mice (PBS + DSS) compared to the healthy control mice (PBS). Protegrin-treated groups (ProPG + DSS, Cathelin + DSS, PG-1 + DSS) appeared to prevent the shortening of the colon length and the decrease in colon weight due to DSS, in which the colon length and weight were comparable to those of the healthy PBS control (Figure 3C–E; *P* < 0.05).

### Histopathological Analysis of DSS-Induced Colitis

**FIGURE 4:**
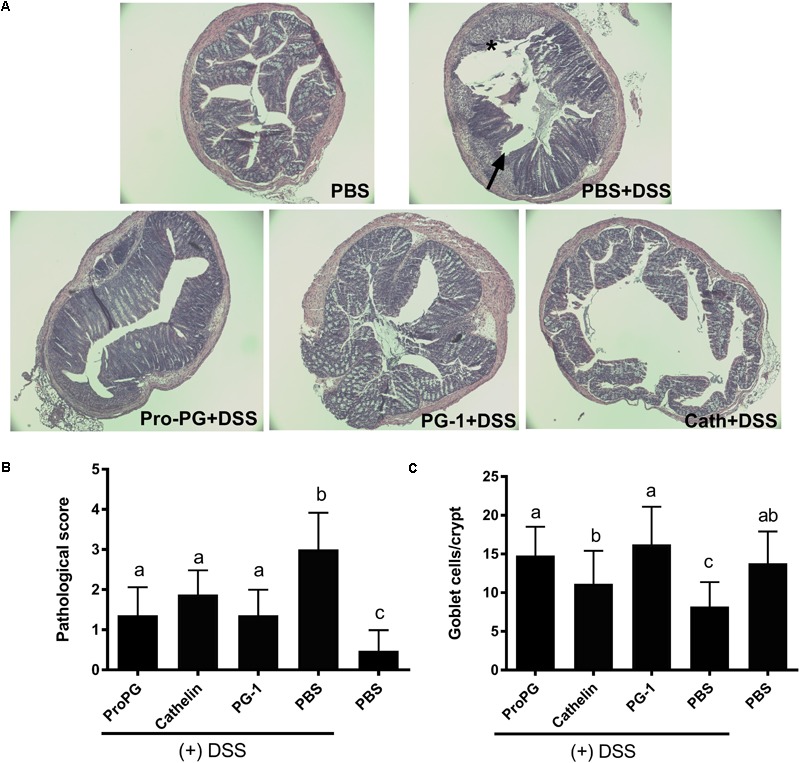
Protegrin improves colonic histomorphology in DSS-induced colitis in mice. **(A)** DSS-induced mucosal crypt loss (arrow), erosion and inflammation. Protegrin-treated groups (ProPG and mPG-1) have less mucosal erosion (^∗^) and submucosal inflammation (arrow) when compared to the DSS-only treatment group (PBS + DSS); **(B)** pathologic score. Surface epithelial loss, inflammatory cell infiltration into the mucosa, and crypt destruction were scored (0 for no change; 1 for localized and mild, 2 for localized and moderate; 3 for extensive and moderate; 4 for extensive and severe). The pathologic score is the sum of the scores for these three parameters. **(C)** Goblet cell count (goblet cells per crypt). Data represent the mean ± SEM of three mice per treatment. Different letter superscripts denote significant difference between groups (*P* < 0.05) calculated using a one-way ANOVA with a *post hoc* Tukey test.

Loss of crypts and evidence of inflammation were absent in the PBS group (healthy control) mouse colon sections stained with H&E (Figure 4A). In contrast, colon tissue from the PBS + DSS group showed extensive areas of mucosa with loss of crypts, erosion, and inflammation. The administration of protegrin (ProPG + DSS, Cathelin + DSS, PG-1 + DSS groups) altered the appearance of the mucosa, reverting it closer to the healthy control (PBS) group with decreased inflammation and epithelial erosion. The histological score was sevenfold higher in the PBS + DSS group compared to the healthy (PBS) control group (Figure 4B). With protegrin treatments, the histological scores were significantly lower than the negative control (PBS + DSS), although still 2.6-fold (ProPG + DSS), 3.6-fold (Cathelin + DSS) and 2.5-fold (PG-1 + DSS) higher than the non-DSS (PBS) control. Histological analysis showed a 42% decrease in mucus-laden goblet cell number in the PBS + DSS group compared to the healthy (PBS) control group (Figure 4C) (*P* < 0.05). ProPG + DSS and PG-1 + DSS groups had similar number of mucus-laden goblet cells as the healthy (PBS) control. The Cathelin + DSS group had 28% higher number of mucus-laden goblet cells than the PBS + DSS group (*P* < 0.05) and had 18% lower number of mucus-laden goblet cells than the healthy PBS control, although this was not statistically significant.

### Influence of Protegrin on Expression of Mediators of Inflammation and Tissue Repair

**FIGURE 5:**
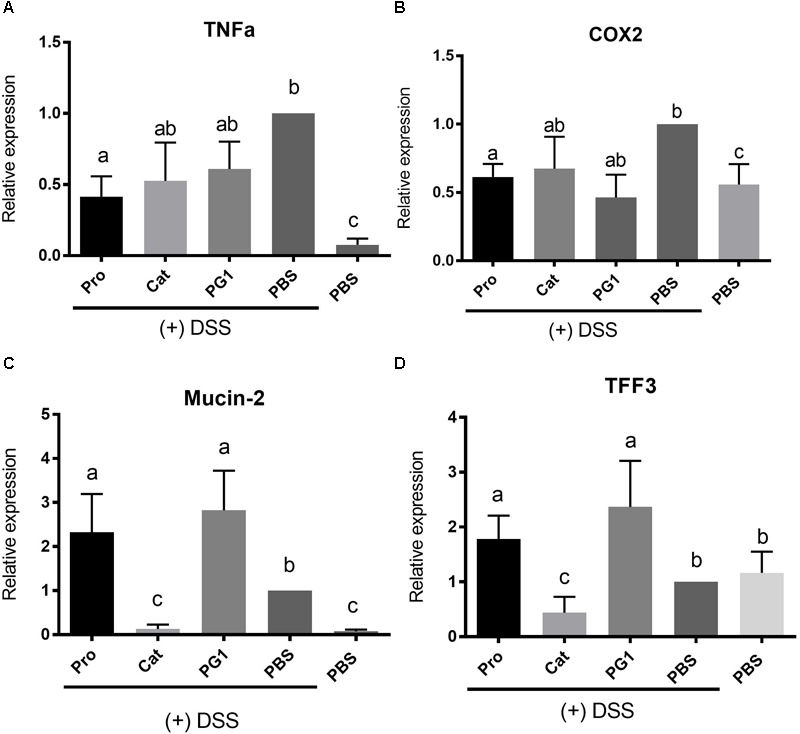
Effect of protegrin administration on modulating gene expression of inflammatory mediators TNFα **(A)** and Cox-2 **(B)** and tissue-protection factors mucin-2 **(C)** and trefoil factor (TFF)-3 **(D)** in mice challenged with DSS. Colonic expression level of these genes was determined by quantitative RT-PCR and values were normalized against the reference gene β-actin. Data represent the mean ± SEM of six mice per treatment. Different letter superscripts denote significant difference between groups (*P* < 0.05) calculated using a one-way ANOVA with a *post hoc* Tukey test.

Previous studies have shown that altered levels of inflammatory cytokines and tissue-repair factors can correlate with the severity of colitis (Williams et al., 2001; Faure et al., 2003; Yan et al., 2009). We employed RT-qPCR to examine the expression of factors involved in inflammation and tissue repair, which may play a role in modulating colitis under DSS treatment. As shown in Figure 5, DSS treatment (PBS + DSS group) significantly increased the production of the inflammatory mediators TNFα and COX-2 in comparison to healthy PBS control by 20- and 2-fold, respectively (*P* < 0.05). ProPG treatment (ProPG + DSS group) resulted in significantly lower (*P* < 0.05) TNFα level in comparison to the PBS + DSS colitis control group, although it was still significantly (*P* < 0.05) greater than the non-DSS (PBS) control. Cathelin and PG-1 administration did not result in significant reduction of TNFα level in comparison to the PBS + DSS group. ProPG- and PG-1-treated mice administered DSS had similar COX-2 expression levels as the healthy PBS control (Figure 5), whereas Cathelin treatment (Cathelin + DSS) did not significantly reduce COX-2 levels compared to the PBS + DSS control group. These results suggest protegrin can modulate inflammatory mediators associated with DSS-induced colitis.

Mediators for mucosal protection and tissue repair, specifically mucin-2 (MUC-2) and trefoil factor (TFF3), respectively, were also measured. MUC-2 expression was increased in the PBS + DSS group compared to the healthy PBS control, likely due to a self-protective response. Interestingly, cathelin treatment (Cathelin + DSS) decreased MUC2 expression to a level comparable to the healthy control (Figure 5). MUC-2 expression level was increased in ProPG + DSS and PG-1 + DSS groups by 2.4- and 2.7-fold relative to the PBS + DSS control (*P* < 0.05; Figure 5). Similar to MUC-2, TFF3 expression level was upregulated by 2- and 2.7-fold in the ProPG + DSS and PG-1 + DSS groups relative to the PBS + DSS control (Figure 5). TFF3 expression did not differ significantly among the cathelin + DSS, PBS + DSS, and healthy PBS control groups.

### Effect of Cathelicidin Protegrin on Serum Metabolite Profile in Mice With Colitis

Previous studies have shown distinct differences in the metabolite profile of healthy individuals and those with IBD (Marchesi et al., 2007; Williams et al., 2009). To determine the changes in endogenous metabolites induced by DSS and protegrin treatment, serum samples were collected from mice at Day 10 of DSS-induction for untargeted detection of metabolites including amino acids, biogenic amines, acylcarnitines, and phospho- and sphingo-lipids.

**FIGURE 6:**
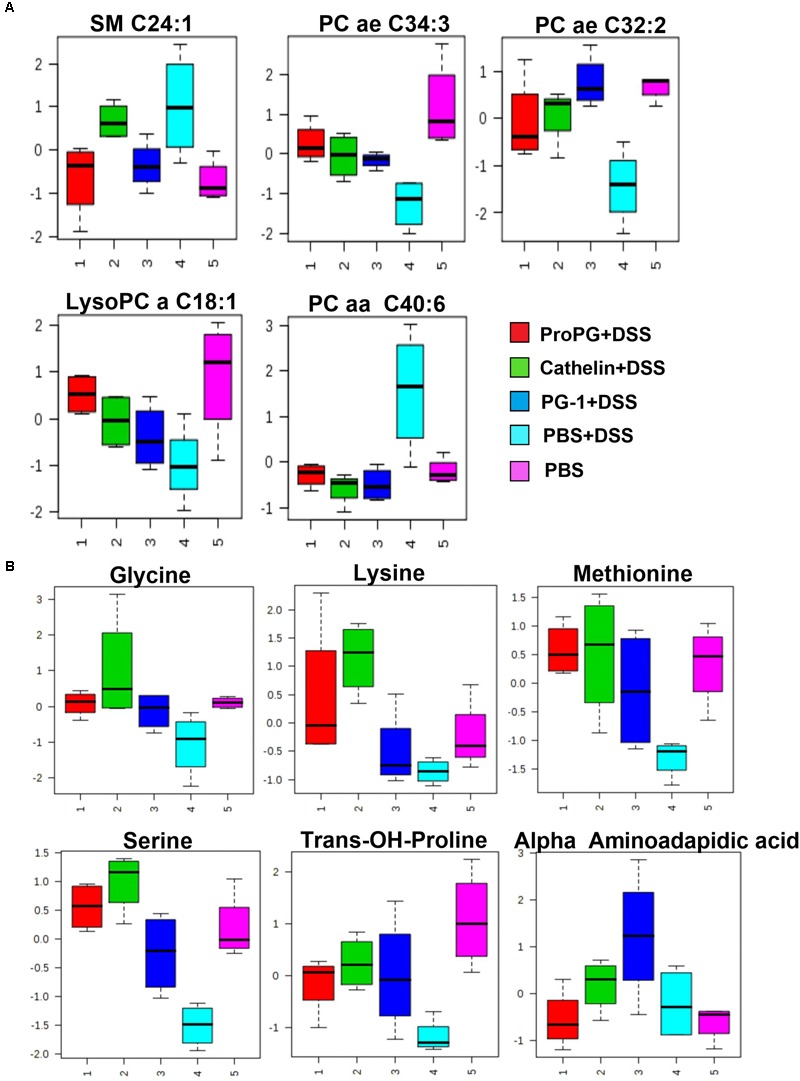
Influence of PGs on serum sphingomyelin and glycerophospholipids **(A)** and amino acid **(B)** metabolites in DSS-induced colitis control. SM (Sphingomyelin), PC aa (phosphatidylcholine diacyl), PC ae (phosphatidylcholine acyl-alkyl), and lysoPC (lysoPhosphatidylcholine acyl). Data represent the mean ± SEM of four mice per treatment.

Univariate ANOVA (Bartel et al., 2013) was performed to analyze metabolites, and reduce the number of measured metabolites to only those that showed the strongest response under the investigated treatment conditions. Of the ∼200 metabolites screened, a total of 11 metabolites were identified to be significantly different between the PBS + DSS and healthy PBS control group (Figure 6). As shown in Figure 6A, serum levels of sphingomyelin (SM C24:1) and phosphatidylcholine acyl (PC aa C40:6) were increased in the colitis group (PBS + DSS), while phosphatidylcholine acyl-akyl (PC ae C34:3, PC ae C32:3) and lysoPhosphatidylcholine acyl (LysoPC a C18:1) were lower in the PBS + DSS group compared to the healthy PBS control. Glycine, lysine, methionine, serine, *trans*-OH-proline, and α-aminoadipic acid levels were also lower in the PBS + DSS control compared to the healthy PBS control group (Figure 6B). The treatment with protegrins (ProPG + DSS, cathelin + DSS, and PG-1 + DSS) appeared to revert these metabolite levels closer to those in the healthy control (PBS) group.

## Discussion

Endogenous AMPs, also referred to as host defense peptides, play an important role in innate immunity. In recent years, human and mouse cathelicidins have been shown to influence inflammation and tissue/wound repair. These processes regulate leukocyte chemotaxis (Verbanac et al., 1993; Scott et al., 2002; Tjabringa et al., 2003; Kurosaka et al., 2005; Nagaoka et al., 2006), and stimulate angiogenesis, cell migration, and proliferation (Tokumaru et al., 2005; Zanetti, 2005; Kajiya et al., 2010; Wuerth and Hancock, 2011; Yeung et al., 2011). In IBD, the colonic expression of cathelicidin is altered with increased expression in ulcerative colitis patients compared to the non-inflamed control mucosa, suggesting a potential role of cathelicidin in modulating intestinal inflammation (Schauber et al., 2006). In the present study, the potential protective effects of protegrins in an acute mouse colitis model were investigated.

Dextran sodium sulfate-induced colitis is a common model in which the chemical is orally administered to disrupt the intestinal epithelial barrier causing acute colitis with bloody stools and diarrhea (Maxwell et al., 2009). In previous studies (Vowinkel et al., 2004; Yan et al., 2009), mice treated with DSS in the drinking water showed BW loss, ruffled coat appearance and decreased activity level. Histological lesions were also evident and enough to compromise normal intestinal function (e.g., absorption) to result in BW loss. In our current *in vivo* study, oral administration of the pro-form (proPG-1), the pro-region (cathelin-like domain), and the mature PG-1 protected the mice from developing of DSS-induced colitis. Specifically, the protegrin treatments: (1) partially prevented BW loss and colon shortening associated with DSS treatment; (2) improved overall DAI score compared to the colitis (PBS + DSS) control group; (3) reduced histologic lesions with milder epithelial damage and decreased inflammation; (4) increased goblet cell number; (5) decreased inflammatory response; and (6) altered the metabolomic profile pattern to return it closer to the healthy control (PBS) group pattern.

Mature PG-1 and cathelin treatment resulted in similar effects on intestinal weight, length and histological scoring. Of the three forms of protegrin, the pro-form protegrin (ProPG) appeared to have more protective effects compared to mature PG-1 and cathelin, perhaps due to the synergistic effect of the cathelin pro-piece and the mature form. Within the recombinant ProPG, an enterokinase (EK) cleavage site (DDDDK) replaced the native neutrophil elastase site between the cathelin domain and the mature PG-1 domain, allowing mature PG1 release by enzymatic cleavage.

Goblet cells in the intestine synthesize mucin and trefoil factor peptides, and it is known that goblet cell number can decrease in the inflamed mucosa of mouse colitis models and in human IBD (Einerhand et al., 2002; Mizoguchi and Mizoguchi, 2008). Goblet cell number is one of the indicators of gut health. In response to stressors, changes in intestinal mucin level can occur, influencing their protective function in the intestinal tract. In the current study, ProPG and PG-1 treatment prevented the reduction of goblet cell number associated with DSS treatment, and this was reflected in a healthy appearance of the intestine.

Mucin-2 is one of the proteins secreted by goblet cells, serving a protective role in the intestine (Johansson and Hansson, 2013), and trefoil factor (TFF)-3 is a tissue repair factor involved in the regulation of epithelial restitution and epithelial cell migration (Hoffmann, 2005). The elevated expression level of Muc-2 and TFF3 in groups treated with ProPG and PG-1 corresponded to a higher mucus-laden goblet cell number in comparison to the PBS + DSS control. It is possible that the DSS colitis stimulates increased Muc2 expression in an attempt to protect the intestine. The up-regulation of Muc-2 in the PBS-DSS group compared to the PBS non-DSS group is consistent to this interpretation. An increase in Muc-2 and TFF3 expression could contribute to protection and enhanced tissue repair in colitis, resulting in improved intestinal histological scores and improved clinical performance observed in these two groups. The protection may also be influenced by intestinal commensal flora, which might have been modulated by protegrin. The heterogeneity in the composition of microbial communities (microbiota) has been implicated in the pathogenesis of IBD (Round and Mazmanian, 2009; Matsuoka and Kanai, 2015).

Compared to the ProPG and PG-1 treatments, cathelin treatment resulted in a much lower Muc-2 and TFF-3 expression. The lower Muc-2 expression corresponds to numbers of goblet cells that were higher than the PBS + DSS group and similar to the healthy PBS control group, and improved intestinal histologic scores compared to the PBS + DSS group. These observed trends suggest that the mature PG-1 domain may partially act through the goblet cell, either directly or indirectly, to increase expression of TFF3 and Muc-2 in this diseased model. Treatment with only the cathelin domain still showed overall protective effects in the intestine, resulting in improved DAI similar to that for ProPG and PG-1 treatments. However, it may act through other pathways rather than just regulating goblet cell proliferation and its associated TFF3 and Muc-2 expression. In terms of preventing intestinal weight loss, suppression of TNFα expression from ProPG treatment resulted in further enhanced protective effect compared to either PG-1 and the cathelin domain, suggesting there may be an additive or synergistic effect between the cathelin domain and mature PG1.

This is the first study on the function of proPG-1 and its cathelin domain *in vivo*. While recent work has shed light on potential molecular mechanisms of PG-1 in a cellular model, the *in vivo* effects have not been investigated previously. In a porcine enterocyte cell model, it appears that PG-1 activated the Insulin-Like Growth Factor 1 Receptor (IGF1R) pathway (Penney and Li, 2018). The IGF1R pathway has been linked to immune system modulation as well as cell migration; both of these phenomena were found to be induced by treatment with PG-1 (Penney and Li, 2018). There is no previous knowledge with regard to the *in vivo* functions of different forms of the peptide. Our data revealed similar intestinal anti-inflammatory effects by pro-form PG-1, mature PG-1 and the cathelin domain for most of the inflammatory parameters measured in the study.

Previous work on the PG-3 cathelin domain revealed that the cathelin domain plays a role in activating cathepsin L (Zhu et al., 2008). Cathepsin L is involved in antigen presentation in the early events of the immunological response to infection (Beers et al., 2003). This suggests the protegrin cathelin domain may link innate and adaptive immunity via its activation of cathepsin L (Zhu et al., 2008). In IBD patients, the dysregulation of the innate and adaptive immune pathways can contribute to the abnormal intestinal inflammatory response (Geremia et al., 2014). Contrary to the PG-3 cathelin domain, the pro-piece of the proform hCAP-18 (human cathelicidin) inhibits cathepsin L activation. This has led to the suggestion of a potential role for hCAP-18 in preventing cysteine-proteinase-mediated tissue damage during inflammation (Zaiou et al., 2003). From an evolutionary perspective, this functional divergence of the conserved cathelin-like domain family between the human and the pig may be due to positive selection where the protegrin cathelin-like domain has developed or maintained other functions that are unrelated to modulating cathepsin L activity (Zhu, 2008). For future studies, it would be of interest to identify the mechanism of the protegrin cathelin-like domain involved in suppressing the effects of DSS-induced colitis that we have observed.

To further discriminate between the diseased and healthy mice phenotypes, quantitative metabolomic profiling was used to investigate biomarker patterns. In this study, the metabolomic profile of serum distinguished the healthy control mice from mice with DSS-induced colitis. In the DSS-induced colitis group, serum levels of sphingomyelin (SM C24:1) and phosphatidylcholine acyl (PC aa C40:6) were increased compared to the healthy PBS control, while phosphatidylcholine acyl-akyl (PC ae C34:3, PC ae C32:3) and lysoPhosphatidylcholine acyl (LysoPC a C18:1) were decreased. Amino acids including glycine, lysine, methionine, serine, *trans*-OH-proline, and α-aminoadipic acid levels were decreased in the PBS + DSS control compared to the healthy PBS control group. These results indicate metabolomic profiling can be used to distinguish differences between mice that are healthy and those with colitis, to potentially determine diagnostic biomarkers of disease severity. The treatment with protegrins (ProPG, cathelin, and PG-1 + DSS) appeared to revert these metabolite levels closer to the healthy control (PBS) group level, suggesting the intestinal disease severity decreases with protegrin treatment. Whether or not protegrins have a direct or a specific effect on these metabolites remains unknown and speculative.

These lipid metabolite compounds are ubiquitous building blocks for eukaryotic cell membranes and have importance in cell signaling, epithelial integrity, and pathological processes including inflammation (Maceyka and Spiegel, 2014). The breakdown of sphingomyelin to ceramide and sphingosine-1-phosphate has been suggested, by several studies, to have an integral role in inflammation (reviewed in Nixon, 2009). In our study, the DSS-colitis-induced sphingomyelin levels (SM C24:1) were decreased by Pro-PG-1 and PG-1 (Figure 6A) suggesting a decrease in inflammation after treatments. Our finding is consistent with the report that sphingomyelin synthase 2 (SMS2) deficiency inhibited the initiation of DSS-induced colon inflammation by regulating the proinflammatory immune response via inhibition of gene expression (Ohnishi et al., 2017). It is possible that PG-1 treatment resulted in suppression of SMS2 expression and thus decrease SM level, although the pathways involved in this regulation is currently unclear.

In this respect, the sphingomyelin level in the diseased (PBS + DSS) mice and cathelin-treated mice were similar, and higher than the healthy control mice (Figure 6A). Ceramide can be involved in the activation of pro-inflammatory transcription factors, leading to the production of inflammatory mediators including prostaglandins (COX-2, PGE2) and TNFα (Nixon, 2009; Maceyka and Spiegel, 2014).

Glycerophospholipid metabolites (PC ae C34:3, PC ae C32:2, PC aa C40:6, lysoPC a18:1) were significantly different from the diseased group (PBS + DSS). Although the specific mechanism of each of these metabolites in inflammation is currently unclear, patients with ulcerative colitis have been shown to have altered levels of phosphatidylcholine (Treede et al., 2007). Patients with ulcerative colitis have been shown to have altered levels of phosphatidylcholine (Treede et al., 2007). In this study, the phosphatidylcholine levels in all three protegrin-treated groups were reverted closer to the healthy control (PBS) group level, which correlated with the clinical health status of these mice. In addition, amino acids (glycine, lysine, methionine, serine, *trans*-OH-proline) were decreased in the serum of PBS + DSS mice. This may be due to lower food intake, anorexia and/or protein loss (due to mucosal erosions in the colon) associated with this experimental colitis model. Furthermore, protein catabolism is known to occur in inflammatory conditions where a release of amino acids from muscle tissue can provide substrates for proteins of the immune system (Grimble, 2001; Schicho et al., 2010). A decreased level of serum amino acids can be the result of an increased demand for them during inflammation (Schicho et al., 2010). This was observed especially in the PBS-DSS diseased control group, where the amino acid profile is generally lower compared to the protegrin treatment and healthy control groups. Similar to the glycerophospholipid metabolites, the amino acid profile trend correlated with the clinical health status of these mice.

## Conclusion

In summary, the current study demonstrated the potential protective effects of protegrin in an acute mouse colitis model. To our knowledge, this is the first report comparing the protective effects of recombinant PG-1 in its pro-, cathelin-, and mature-form. All three forms attenuated the significant BW loss associated with DSS-induced colitis, though the mechanism of action of each form remains unknown. Further histologic, gene expression and metabolomic analyses indicated that each form exerts its effect at varying degrees, bringing values closer to the healthy (PBS) control levels. Recombinant ProPG and mature PG-1 results were similar to each other, while the cathelin-like peptide results differed significantly from those of ProPG and PG-1. This may suggest differences in the mechanism of action of the cathelin domain. The mechanisms by which protegrins regulate expression of the inflammatory gene transcripts and metabolites remained to be further elucidated. Our findings provide insights into a novel tissue protective role of protegrin, and may lead to therapeutic potential of this antimicrobial peptide for IBD.

## Author Contributions

EH contributed to project design, data acquisition and analysis, and interpretation and wrote the manuscript. JC and JL contributed to project design and data interpretation and critically revised the manuscript. JP contributed to figure configuration, data interpretation, critical revision of manuscript, and finalized and submitted the final approved version.

## Conflict of Interest Statement

The authors declare that the research was conducted in the absence of any commercial or financial relationships that could be construed as a potential conflict of interest.

## Footnotes

1 http://www.metaboanalyst.ca
